# High incidence of TB at a psychiatric hospital

**DOI:** 10.5588/ijtldopen.24.0684

**Published:** 2025-06-13

**Authors:** X. Li, X. Pang, C. Mu, F. Zhang, C. Wang, G. Zhang

**Affiliations:** Tianjin Center for Tuberculosis Control, Tianjin 300011, China.

**Keywords:** tuberculosis, China, whole-genome sequencing, epidemiological investigation, infection control

## Abstract

**BACKGROUND:**

Individuals with mental illness are susceptible to TB. This study aimed to investigate a TB epidemic in a male psychiatric hospital to explore its potential for institutional spread.

**METHODS:**

From April 2022 to March 2023, 19 TB cases were diagnosed in a male psychiatric hospital. Epidemiological investigations and screening of close contacts were carried out.

**RESULTS:**

A total of 400 psychiatric patients and 160 staff members were screened for TB. The overall positive rate was 37.15%, with 43.09% among patients and 22.93% among staff. Ultimately, a total of 17 psychiatric patients (including 2 index cases, 5 active cases, and 11 confirmed cases) and 2 staff (1 active case and 1 confirmed case) were diagnosed. The overall attack rate was 3.36%, with 4.20% in patients and 1.24% in staff. Whole-genome sequencing revealed that 3 drug-resistant patients from a third Department had mutations at two loci (rpoB and rpsL) with fewer than 6 SNPs.

**CONCLUSION:**

Strengthening surveillance and conducting comprehensive epidemiological investigations for any occurrence of two or more TB cases is of utmost importance. Additionally, enhancing diagnostic capabilities and ensuring strict adherence to infection control protocols during patient care are essential measures to prevent TB outbreak.

TB is one of the top ten causes of death globally. In 2023, it is estimated that there were 741,000 new TB patients in China, with an estimated incidence of 52 per 100,000.^1^ Certain populations, including students,^2^ the homeless,^3^ prisoners,^4^ and mental patients,^5–7^ are at an increased risk of TB infection and transmission due to being in relatively overcrowded conditions. The TB prevalence of mental patients is 3.6–6.4 times higher than that in the general population.^8,9^ Moreover, TB has been reported to be a leading cause of infectious diseases in mental hospitals, and accounts for approximately 35% of all infectious cases, ranking it among the top two infectious diseases in such settings.^10^ This underscores the importance of robust TB prevention, detection and management strategies in mental health facilities.

The prevention, treatment, control and management of patients suffering from both mental illness and TB have presented challenges. Both diseases exert a severe impact on health, and therapeutic drugs for both conditions can cause liver and kidney damaged. Thus, the treatment of comorbidity is more susceptible to adverse drug reactions. Notably, a relatively high rate (63.9%) of adverse reactions has been witnessed in schizophrenic patients undergoing anti-TB therapy.^11^ In the event that an active TB patient fails to be detected promptly or is treated without achieving the desired effectiveness, there exists a high likelihood of triggering clustered epidemics or outbreaks.^12^ Here, we report on a TB outbreak that occurred in a psychiatric hospital. Field epidemiological investigations and screening of close contacts were conducted and revealed lessons that deserve reflection. The aim of this study is to assess the prevalence of Latent TB infection (LTBI) and TB disease at the psychiatric hospital to evaluate the possibility of intra-institutional transmission.

## METHODS

Before the outbreak was confirmed, the staff at the Center for Tuberculosis Control monitored two TB cases in the same psychiatric hospital on May 31, 2022. Subsequently, they contacted the local Center for Disease Control and Prevention (CDC) and psychiatric hospital to investigate the details of the two cases. The first index case, a 35-year-old male patient with rifampicin-resistant TB (RR-TB), was reported on January 24th. Four days later, another 36-year-old male patient with smear-negative TB was reported.

### TB classification

The diagnosis of TB was based on the Diagnosis of Tuberculosis (WS 288-2017).^13^ A bacteriologically confirmed pulmonary TB (PTB) case was defined as a patient with any positive result shown by sputum smear microscopy, culture or a WHO-approved nucleic acid amplification test, such as Xpert. A patient diagnosed in the absence of the above-mentioned evidence was defined as a clinically diagnosed PTB patient.

Cases should either have sputum that is smear-negative but culture-positive for *Mycobacterium tuberculosis* (M. tb) or meet the following diagnostic criteria: decision by a clinician to treat with a full course of anti-TB therapy; and radiographic abnormalities consistent with active PTB.^14^ For patients with a state of persistent immune response to stimulation by antigens with no evidence of clinically manifest active TB, either a tuberculin skin test (TST) or interferon-gamma release assay (IGRA) can be used to test for LTBI. Anyone meeting one of the following conditions could be diagnosed as a suspected case: (1) When a chest radiograph reveals a lesion that is suspected to be PTB; (2) Having suspicious symptoms of TB; (3) For other atypical symptoms accompanied by a moderately positive purified protein derivative (PPD) result (induration diameter≥10 mm) or a positive IGRA test. Preventive therapy was recommended for those with a strong positive PPD alone (induration diameter≥15 mm, or with double ring, blister, necrosis and lymphangitis reactions).^15^

Those who have accumulated contact with a TB patient for 40 hours within six months before the onset of illness, or accumulated contact for 8 hours or more in a day was classified as a close contact. Given that psychiatric patients within a department rotate their sleeping arrangements every three months and share living and dining spaces around the clock, the department was designated as the smallest unit for contact tracing. All patients from the same department as the confirmed cases, as well as all hospital staff, were classified as close contacts.

### TST testing

TST was performed by a trained doctor/nurse, injecting intradermally 0.1 ml (2 IU) of PPD (Chengdu Biological Products Research Co.,Ltd., Chengdu, China) into the inner surface of the left forearm. An experienced physician measured the transverse induration at the TST site 48-72 h after injection.^16, 17^ TST results were categorised into ≥5, ≥10 and ≥15 mm induration reactions. ≥10 mm was clasified as positive and ≥15 mm was strong positive.

### Whole gene sequencing (WGS)

DNA was extracted using the modified CTAB method.^18^ The entire library preparation process was completed through steps such as end repair, A-tailing, addition of sequencing adapters, purification, and PCR amplification. After the library was constructed, it was initially quantified using Qubit 2.0, diluted, and then the insert fragments of the library were detected using Agilent 2100. The effective concentration of the library was accurately quantified using the Q-PCR method. Different libraries were pooled into the flowcell according to the effective concentration and the required amount of sequencing data, and clusters were formed using cBOT before sequencing on the Illumina high-throughput sequencing platform NovaSeq 6000. After sequencing, cleaned reads were aligned to the M. tb reference genome (H37Rv) and processed using SAMtools. Variants were screened based on quality metrics, allele frequencies, and functional annotations, and the data was subjected to genetic distance analysis, drug resistance analysis and homology analysis. Based on previous studies, isolates with a pairwise genetic distance of less than 12 SNPs were defined homologous.^19,20^

### Case detection and epidemiological investigation

After confirming the initial two index cases, the staff of the Center for Tuberculosis Control immediately conducted field epidemiological investigations and close contact screening. The CDC personnel scoured the TB Information and Management System (TBIMS) and interviewed staff at the psychiatric hospital to identify TB cases. Simultaneously, all chest X-ray examinations conducted in the psychiatric hospital since 2022 were retrospectively reviewed by experts with more than 10 years of clinical experience. Psychiatric patients and staffs were screened using a standardized questionnaire, TST and chest X-ray examinations. Individuals with suspicious TB symptoms, strong positive PPD reactions, or abnormal chest X-rays were referred for further clinical assessment. For those individuals with a positive TST, an additional chest X-ray examination was recommended 3 months later. This was aimed at identifying those individuals who had contracted TB but were still within the window period during the initial screening process.

All statistical analysis were conducted using SPSS software (version 16.0, SPSS Inc., Chicago, USA). The categorical data were compared by the χ^2^ test or Fisher’s exact test as appropriate, and t-tests were used for continuous variables. P-values < 0.05 was considered statistically significant.

The study was approved by the Ethics Committee of Tianjin Center for Tuberculosis Control. Written informed consents were obtained from the diagnosed PTB patients.

## RESULTS

The outbreak occurred at a male psychiatric hospital with a total of 405 psychiatric patients and 161 staff members. The facility consists of two main buildings (labeled as A and B), with four floors. Each building contains four departments. The 4th floor of building A is Department 7, which mainly serves as an isolation ward for cases with infectious diseases and a buffer ward for newly admitted patients. Each ward is equipped with 2–6 beds and a private bathroom, the rooms lack doors. Ventilation is limited as each room has a single window that can only be opened less than 12 cm. The hospital depends on central air conditioning for climate control. Patients are not assigned to the same bed and rotate on average every three months. The majority of psychiatric patients exhibit a lack of hygiene awareness and often misuse and misplace personal hygiene items. The hospital implements a zoned isolation management system, with each department being relatively independent while sharing common living and resting areas.

### Index cases

The two index patients were a 35-year-old male psychiatric patient with RR-TB in Department 3 and a 36-year-old male psychiatric patient, etiologically negative, in Department 1. Both were referred to a specialized TB hospital for further evaluation after abnormal findings on their chest X-rays during routine in-hospital physical examinations, even though there were no overt PTB symptoms. They were diagnosed on January 24 and January 28, respectively, in accordance with the ‘Standard of Diagnosis for Pulmonary Tuberculosis (WS 288-2017)’.^13^ Following their diagnosis, they were admitted to isolation wards in Department 7 to receive directly observed treatment, short-course (DOTS) anti-TB therapy.

### Epidemiologic investigation and contact screening

**Figure 1.**
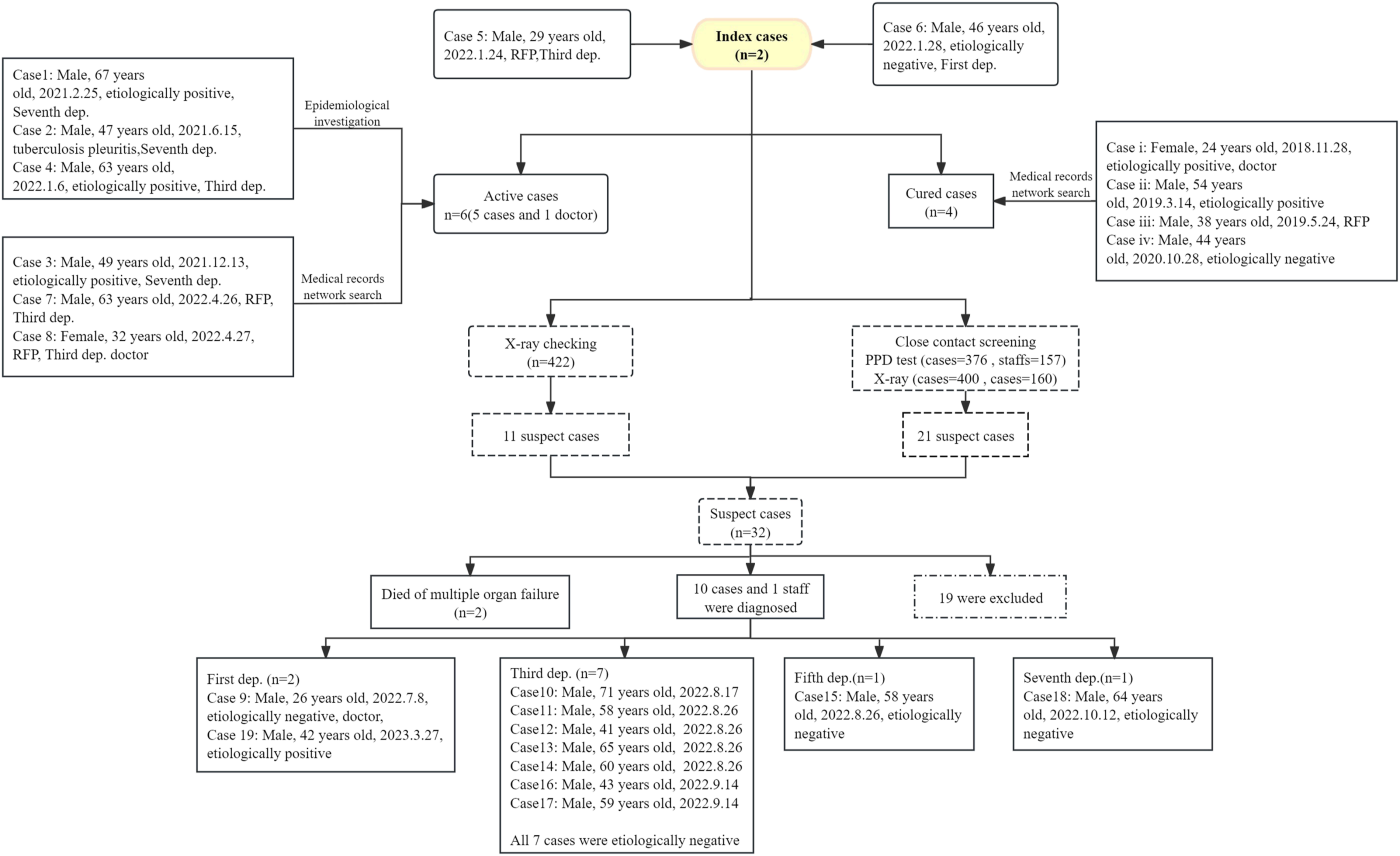
Diagnosis and exclusion of all suspected cases found during the epidemiological investigation and screening of close contacts in a psychiatric hospital. PPD = purified protein derivative.

After discovering the two index cases, there was a comprehensive review of the national TB report system and on-site epidemiological investigation. It was found that between November 2018 to October 2020, 4 TB cases were confirmed (2 positive, 1 negative, and a RR-TB case). All were cured without recurrence. Currently, there are 6 TB cases in the hospital, 5 are patients (3 positive, 1 RR-TB and 1 case of tuberculous pleurisy), and 1 case is a medical staff member (female, with RR-TB). After reviewing 422 chest radiographs from January 1, 2022 to June 7, 2022, 11 suspected TB cases were identified (for 24 patients, the results could not be read). A total of 533 contacts (376 patients and 157 staff members) had PPD testing results; the overall strong positive rate was 37.15% (43.09% among patients and 22.93% among staff members). Department 3 had the highest positive PPD rate (56.38%). 560 individuals underwent chest X-ray examinations (160 staff members and 400 patients), with a total of 44 abnormalities (20 patients and 24 staff members).The process and results are shown in Figure 1 and Table 1.

**Table 1.** Epidemiological investigation and screening for close contacts.

Depts	Staff					Psychiatric patients					Total		
	PPD		X-ray		Suspect patients n (%)	PPD		X-ray		Suspect patients n (%)	Strong positive PPD n (%)	Abnormal X-ray n (%)	Suspect patients n (%)
	Screening	Strong positive n (%)	Screening	Abnormal n (%)		Screening	Strong positive n (%)	Screening	Abnormal n (%)				
First	25	16 (64)	25	12 (48)	1 (4)	117	57(48.72)	118	1 (0.85)	6 (5.08)	73 (51.41)	13 (9.09)	7 (4.9)
Third	26	1 (3.85)	26	3 (11.54)	0 (0)	94	53 (56.38)	108	6 (5.56)	11 (10.19)	54 (45)	9 (6.72)	11 (8.21)
Fifth	26	6 (23.08)	26	5 (19.23)	0 (0)	116	34 (29.31)	117	2 (1.71)	2 (1.71)	40 (28.17)	7 (4.9)	2 (1.4)
Seventh	29	11 (37.93)	29	4 (13.79)	0 (0)	34	12 (35.29)	42	11 (26.19)	12 (28.57)	23 (36.51)	15 (21.13)	12 (16.9)
Others	51	2 (3.92)	54	0 (0)	0 (0)	15	6 (40)	15	0 (0)	0 (0)	8 (12.12)	0 (0)	0 (0)
Total	157^A^	36 (22.93)	160	24 (15)	1 (0.63)	376^B^	162 (43.09)	400	20 (5)	31 (7.75)	198 (37.15)	44 (7.86)	32 (5.71)

A Three patients received COVID-19 vaccine and did not undergo PPD;

B For 24 patients, the results could not be read.

PPD = purified protein derivative.

**Figure 2.**
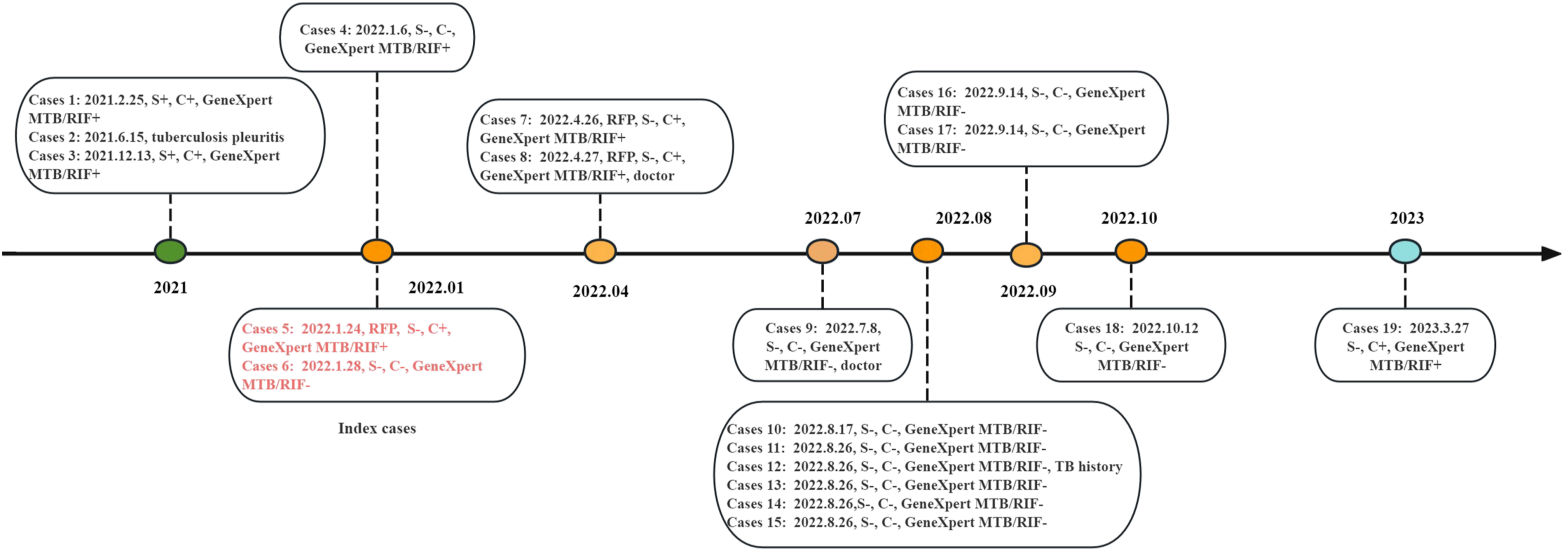
Timeline of confirmed TB cases in a psychiatric hospital, China. S+ = smear-positive; C+ = culture-positive; Smear− = smear-negative; C− = culture-negative; RR-TB = rifampicin-resistant TB; GeneXpert MTB/RIF− = GeneXpert MTB/RIF negative; GeneXpert MTB/RIF+ = GeneXpert MTB/ RIF positive.

In total, 32 suspected TB patients were identified. Unfortunately, 2 of these suspected patients died from multiple organ failure before confirmation. Ultimately, 11 TB cases were confirmed (1 staff member and 10 patients) and the other 19 cases were excluded after a negative sputum test. By March 31, 2023, a total of 19 cases (17 patients and 2 employees) were ascertained as TB cases: 4 were etiologically positive, 11 were etiologically negative, 2 were RR-TB cases, 1 was tuberculous pleurisy and 1 was multidrug- resistant TB (MDR-TB). The average age was 51.8 years (ranging from 26–71 years). The timeline of the outbreak is shown in Figure 2. The overall attack rate was 3.36%, with 4.20% in patients and 1.24% in staff. The incidence rate of psychiatric patients in the Department 3 was 7.30%, followed by those in Department 7 (6.85%, Table 2).

**Table 2.** Department distribution of confirmed TB cases in a male psychiatric hospital, China.

Depts	Patients			Employees			Total		
	TB cases	N	Attack rate No.(%)	TB cases	N	Attack rate No.(%)	TB cases	N	Attack rate No.(%)
First	2	119	1.68	1	25	4.00	3	144	2.08
Third	9	110	8.18	1	27	3.70	10	137	7.30
Fifth	1	117	0.85	0	26	0.00	1	143	0.70
Seventh	5	44	11.36	0	29	0.00	5	73	6.85
Others	0	15	0.00	0	54	0.00	0	69	0.00
Total	17	405	4.20	2	161	1.24	19	566	3.36

To explore potential epidemiological connections among the TB cases, WGS was performed on the four bacteriologically confirmed patients. The WGS results showed the number of SNP differences between the four TB patients was 2–219 (Table 3), the three RR-TB patients (cases 5, 7, and 8) had mutations at two loci (rpoB and rpsL) with SNPs fewer than 6, indicating that their strains belong to the same cluster. Another patient (case 3, Department 7) was not a RR-TB case, and the SNP difference with these three cases exceeded 200. According to the results of WGS and field epidemiological investigation, the three patients were defined as homologous.

**Table 3.** The number of SNP differences observed between the four patients.

SNP-dists	Reference	Case 3	Case 7	Case 8	Case 5
Reference	0	1281	1284	1289	1277
Case 3	1281	0	215	219	217
Case 7	1284	215	0	4	2
Case 8	1289	219	4	0	6
Case 5	1277	217	2	6	0

### Analysis of the outbreak

From January to August 2022, Department 3 consecutively confirmed 9 TB cases. The incidence level was significantly higher than historical levels. The strong positive rate of PPD in Department 3 exceeded that of other departments. Additionally, WGS results indicated that the strains from three RR-TB patients (cases 5, 7, and 8) in Department 3 are genetically related, as they belong to the same cluster. This molecular epidemiological evidence points to intra-departmental transmission of M. tb. The remaining cases were distributed in the 1st, 5th, and 7th Departments. There was no clear epidemiological linkage evidence with other confirmed cases.

## DISCUSSION

We conducted a TB investigation at a psychiatric hospital in China and identified 19 cases. Psychiatric patients are susceptible to infectious disease as they have cognitive impairment and their resistance is decreased when they receive antipsychotic drugs, coupled with long-term insufficient nutritional intake.^21^ In our study, WGS revealed that 3 RR-TB cases from Department 3 were infected with the same strain. This genetic similarity, when corroborated with on-site epidemiological investigations, suggests a potential epidemiological linkage.

The epidemiological investigation revealed several problems.

First, the hospital had not effectively conducted timely epidemiological investigation and close contact screening. This oversight facilitated the intra-departmental spread of M. tb. The results of this study indicate a higher risk of TB development for patients residing in the same department as confirmed cases, compared to those in other departments. A previous study^22^ showed that the positive rate of tuberculin reaction is positively correlated with the degree of contact with infectious sources, and the screening results of our study showed that the positive rate of PPD in Department 3 was higher than that in other departments. Furthermore, WGS data demonstrated genetic homology between patients and medical staff within the same department, implying intra-departmental transmission of the pathogen. According to the results of the field epidemiological investigation, the windows in the psychiatric wards only open to a maximum of 12 cm, and the doors facing the corridors result in inadequate air circulation. Moreover, most psychiatric patients demonstrate poor hygiene practices, frequently mishandling or misplacing their personal hygiene items. Their unrestricted movement between wards without assigned beds significantly increase the likelihood of cross-infection within the ward area.

Second, there is a deficiency in the diagnostic capabilities of the radiology department within the psychiatric hospital. A review of previous chest X-rays identified 11 suspected TB cases, and 6 were ultimately confirmed as TB patients. However, these patients were misdiagnosed as upper respiratory infections, tracheitis, or chronic bronchitis, indicating that psychiatric specialists lack sufficient experience in diagnosing TB and fail to promptly recognize abnormalities on chest X-rays. Furthermore, due to the long-term use of antipsychotic medications and their own psychiatric symptoms, patients are unable to clearly communicate their condition and medical history to doctors, leading to a misdiagnosis rate as high as 78.8%.^23^

Third, current laboratory tests based on sputum may not be suitable for diagnosing active TB in patients with severe mental illness. The results of the close contact screening showed that 24 individuals scratched the site of the PPD injection, making it impossible to read the results; further TB examinations conducted on suspected TB patients revealed that some patients had no sputum or the sputum samples were substandard. The sputum positivity rate was 0, which is consistent with the findings reported in the literature.^24-26^ This indicates that patients with psychiatric disorders struggle to cooperate and may be unable to provide standard sputum samples.

Finally, medical staff did not adhere to standard protective measures while caring for infectious patients. During this outbreak, 2 medical staff were diagnosed, resulting in a morbidity rate of 1.24%. One was found to be homologous with a RR-TB patient. Epidemiological investigation revealed that the infectious disease area and the general ward area shared the same medical staff. Therefore, the occurrence of this outbreak cannot exclude the possibility of M. tb transmission facilitated by medical staff.

A few limitations of this epidemic investigation are worth mentioning, including that we were unable to obtain the 0-month sputum from the 2 RR-TB patients for WGS. Also, the two suspected TB patients who died of respiratory failure were not finally ruled out. However, we were not able to obtain sputum samples, so we do not know if their deaths were related to TB, or if they were related to other TB patients.

As a consequence of this investigation, we recommend that the following measures be taken to prevent and control TB in psychiatric hospitals:

1) pay attention to two or more newly discovered TB cases within six months; 2) conduct timely epidemiological investigation and screening of close contacts; 3) incorporate TB screening (including TST or IGRA, chest X-ray and sputum examination) into routine physical examinations for psychiatric patients; 4) confirmed TB cases should be immediately isolated and undergo rigorous anti-TB chemotherapy; 5) enhance training for medical staff to improve their ability to differentially diagnose PTB, thereby enabling the timely identification of patients; 6) when caring for infectious patients, medical staff must strictly adhere to personal protective measures to prevent cross-infection within the hospital.
